# Longitudinal two-photon imaging in somatosensory cortex of behaving mice reveals dendritic spine formation enhancement by subchronic administration of low-dose ketamine

**DOI:** 10.1038/s41598-018-24933-8

**Published:** 2018-04-24

**Authors:** Evgeny Pryazhnikov, Ekaterina Mugantseva, Plinio Casarotto, Julia Kolikova, Senem Merve Fred, Dmytro Toptunov, Ramil Afzalov, Pirta Hotulainen, Vootele Voikar, Ryan Terry-Lorenzo, Sharon Engel, Sergei Kirov, Eero Castren, Leonard Khiroug

**Affiliations:** 1 Neurotar Ltd, Helsinki, 00790 Finland; 2 Neuroscience Center, University of Helsinki, Helsinki, 00790 Finland; 3 Minerva Foundation Institute for Medical Research, Helsinki, 00290 Finland; 4 Sunovion Pharmaceuticals Inc, Marlborough, MA 01752 USA; 5 Medical College of Georgia at Augusta University, Augusta, 30912 Georgia

## Abstract

Ketamine, a well-known anesthetic, has recently attracted renewed attention as a fast-acting antidepressant. A single dose of ketamine induces rapid synaptogenesis, which may underlie its antidepressant effect. To test whether repeated exposure to ketamine triggers sustained synaptogenesis, we administered a sub-anesthetic dose of ketamine (10 mg/kg i.p.) once-daily for 5 days, and repeatedly imaged dendritic spines of the YFP-expressing pyramidal neurons in somatosensory cortex of awake female mice using *in vivo* two-photon microscopy. We found that the spine formation rate became significantly higher at 72–132 h after the first ketamine injection (but not at 6–24 h), while the rate of elimination of pre-existing spines remained unchanged. In contrast to the net gain of spines observed in ketamine-treated mice, the vehicle-injected control mice exhibited a net loss typical for young-adult animals undergoing synapse pruning. Ketamine-induced spinogenesis was correlated with increased PSD-95 and phosphorylated actin, consistent with formation of new synapses. Moreover, structural synaptic plasticity caused by ketamine was paralleled by a significant improvement in the nest building behavioral assay. Taken together, our data show that subchronic low-dose ketamine induces a sustained shift towards spine formation.

## Introduction

The well-known phencyclidine derivative ketamine has been in medical use as a short-term anesthetic and pain reliever since the 1960s^1,2^. Consumption of ketamine induces vivid sensations with hallucinations leading to a dissociative state, manifest as a sensation of detachment (dissociation) from the environment and from one’s own body. These properties underlie the strong potential of ketamine as a substance of abuse^3,4^ and, subsequently, limit its clinical applicability^5^.

The recent discovery of rapid antidepressant-like action of ketamine^6,7^ has revived clinical interest in this drug. These antidepressant effects are likely related to the newly identified pathways (other than NMDA receptor-mediated), which are activated either by ketamine itself^8^ or by its metabolic by-products^9^.

The synaptogenic potential of ketamine is well documented for frontal/prefrontal cortex^8,10,11^. In a pivotal work, Duman and coauthors have shown that a single intraperitoneal (i.p.) administration of ketamine in mice at the dose of 10 mg/kg rapidly increases synaptic protein levels and dendritic spine number in prefrontal cortex through activation of the mTOR signaling pathway^8^. The same group later demonstrated the BDNF dependence of the ketamine-induced rapid synaptogenesis^10^. Most recently, longitudinal *in vivo* two-photon microscopy was employed to show the stimulating effects of a single i.p. injection of ketamine on dendritic spine formation and density in medial frontal cortex of isoflurane-anesthetized mice^11^.

Despite the insightfulness of the above-mentioned studies, several pivotal questions remain unanswered. First, most studies have only tested acute administration of ketamine. While mimicking the fast and effective treatment of a depression episode with ketamine single dosing^12^, the acute administration protocol prevents implication of these studies’ results to situations when ketamine is administered repeatedly over the course of days or weeks. The reports on effects of subchronic or chronic ketamine administration on plasticity of dendritic spines are scarce^13^. Second, most reports predominantly studied the effects of ketamine on the prefrontal and frontal cortex, due to the depression-centered focus of these studies. It remains, therefore, unknown whether effects of ketamine on synaptic plasticity can be generalized to other cortical areas. Third, some of the previous studies relied on end-point techniques while others used anesthetics (such as isoflurane) during *in vivo* microscopic analysis of spines, which may have significantly affected the outcome of experiments^14,15^. Some depressed patients do not respond to a single dose of ketamine but have been able to achieve additional benefits with additional doses^16^. Therefore, the main purpose of this study was to extend on the previous bodies of work by quantifying the effects of multiple-dose sub-anesthetic ketamine on spine turnover in somatosensory cortex under the best-achievable physiological conditions, i.e., in awake behaving animals.

Two-photon microscopy provides the highest spatial resolution *in vivo* and allows repetitive imaging of the same cells (and subcellular structures) over the course of days or weeks. In this study, we monitored the formation and elimination rates of mature “mushroom”-shaped spines^17^ on apical dendrites of Layer IV-V cortical neurons in the somatosensory cortex. We investigated in awake behaving mice whether subchronic administration of low-dose ketamine induces long-lasting changes in the dendritic spine turnover in somatosensory cortex.

## Methods

All animal procedures were performed in accordance with the University of Helsinki animal care regulations. Local authority (ELÄINKOELAUTAKUNTA-ELLA) approved the animal license (ESAVI/6828/04.10.07/2013) to conduct the procedures described in the study.

### Behavioral tests

The animals (15 female C57BL/6JRccHsd WT mice; 10 week old at the start of the experiment) were group housed. Shortly before the start of behavioral tests mice were moved to type II cages (267 × 207 × 140 mm) with bedding material (aspen chips, Tapvei, Harjumaa, Estonia), and a 2.5 g compressed cotton cloth (50 × 50 mm; Ancare, Bellmore, USA). Mice were single-housed in these cages for the whole duration of behavioral tests. The nest building was scored according to^18^. Briefly, the nest was analyzed for the amount of shredded material and its shape: 1 = nestlet >90% intact; 2 = nestlet 50–90% intact; 3 = nestlet mostly shredded but no identifiable nest site; 4 = identifiable but flat nest; 5 = crater-shaped nest. Following an undisturbed 7 day acclimation period, the nest building score (NBS) was determined and the animals were injected with ketamine (10 mg/kg; n = 8) or vehicle (PBS; 10 ml/kg; n = 7), and the NBS was assessed 22 h after each drug injection (1 daily injection; 4 days) by an observer blinded to the treatment. After each scoring session, the nest material was removed and a new cloth introduced.

During the experimental procedures the animals’ activity was monitored by InfraMot system (TSE, Bad Homburg, Germany), in blocks of 22 h. This system, mounted on top of the cage cover, detects the heat radiating from the animal’s body and its displacement over time. Food and water were available *ad libitum* during the whole experiment, except during cage cleaning and substitution of cotton cloths.

### Animal surgery and treatment

Female 4–10 weeks old B6.Cg-Tg(Thy1-YFP)HJrs/J mice (mouse strain 003782, JaxLab) were anesthetized with ketamine/xylazine and operated for implantation of a cranial window. The cranial window was inserted over the somatosensory cortex at the following coordinates: AP −1.8, ML −2.0 from Bregma. Dental drill (HP4–917, Foredom) was used to remove a round-shaped (d = 4 mm) piece of skull, and the hole in the bone was covered with a round cover glass (d = 5 mm; #72296–05, Electron Microscopy Sciences). Helicopter shaped headplate (model 1; Neurotar) was placed over the cover glass and fixed with dental cement (Rapid Repair; Dentsply) mixed with cyanoacrylate glue (Loctite 401; Henkel) to a skull surface. Based on the transparency of the cranial window 4 weeks after implantation, in total 29 mice were selected for the imaging study and divided randomly in two^2^ groups. Approximately 50% of operated mice were found suitable for imaging. All treatments were by a single i.p. injection at 10 mg/kg per day for 5 consecutive days. The first i.p. injection of ketamine or vehicle was done on day 1, thirty minutes after the baseline imaging session was completed (or at about 2 hours after the start of imaging time 0). Subsequent injections of ketamine or vehicle were performed at the 25 h, 49 h, 73 h and 97 h time points after the first injection. The vehicle consisted of Phosphate Buffered Saline (PBS), containing (in mg/L): 144.00 KH_2_PO_4_, 9000.00 NaCl, 795.00 Na_2_PO_4_. We used the 10 mg/kg i.p. dose, because it is has been associated with synaptogenic effects in rodents^8^ and results in plasma exposures that closely reflect those observed in the clinic^8,19,20^.

### Two-photon imaging

Mice were imaged with the FV1200MPE two-photon microscope (Olympus, Japan) with the 25X water immersion 1.05 NA objective specially designed for *in vivo* two-photon imaging. MaiTai Broad Band DeepSee laser tuned to 900 nm was used for excitation. Emission light was collected using a band pass filter (515–560 nm).

For *in vivo* imaging sessions, awake animals were head-fixed under two-photon microscope using Mobile HomeCage device (Neurotar, Finland). Prior to the imaging sessions, animals were habituated to head fixation in eight(8) two-hour training sessions. Three-dimensional (3D) baseline YFP fluorescence image Z-stacks of the apical dendrites of layer 5 pyramidal neurons in somatosensory cortex were acquired. Although the cell body location of pyramidal neurons was not confirmed in this study, the YFP-H cell line was chosen in part because YFP expression is largely restricted to Layer 5 neurons^21^. Stacks of images were collected with the vertical step of 1 µm (total imaged thickness of stacks: 30–70 µm) with zoom factor three at 800 × 800 or 512 × 512 pixels aspect ratio. Each imaging area spanned 166.7 × 166.7 µm in the field of view. The baseline image stacks (up to 8 stacks per animal) were acquired during the 30–120 min time window before the first injection of vehicle or ketamine. Two subsequent imaging sessions were performed at 24 h and 72 h. The final session was performed either at 120 h (11 mice) or at 144 h (11 mice); the data from this last time point were pooled, the time window binned and the time point designated as the “132 h” point. The total number of imaging sessions per animal was five.

Imaging depth extended to cortical layers 1 and 2; based upon the known Layer 5 neuron morphology, the dendritic spines used for turnover quantification were located predominantly in the apical dendrite tufts. For acquisition, horizontally-oriented dendrites were favored as the x-y resolution of two-photon microscopy is inherently higher than the z resolution. At least 150 dendritic spines were captured at each time point. These spines were collected from the dendrites of multiple different neurons.

### Image Analysis

After acquisition, the images were processed and analyzed using Imaris (Bitplane, United Kingdom) and Fiji/ImageJ (open source image processing package, NIH, USA) software. Shifts of tissue in x, y and z directions were compensated using dedicated plugins in the Fiji software. Spine turnover analysis was performed by independent scientist blinded to the identity of the treatment groups. Individual spines were tracked by visually comparing the spine’s morphology in the stacks of images collected at each of the five time points. Dendritic spines were counted if they protruded for at least 0.5 microns from the dendritic shaft. Only mature, “mushroom”-like spines with head diameters much greater than their neck diameters^17^ were counted. Our aim was to quantify the turnover of 150–200 dendritic spines per animal. Each spine was assigned with a specific code at every time-point: either 1 (present) or 0 (absent). Special attention was paid to exclude pseudo-elimination and/or formation of spines due to shift or rotation of spine in z-direction. For this purpose at least 10 additional slices were visually checked in “up” and “down” direction for every time-point for every spine. At least 125 spines per animal were analyzed for spine density analysis. The data were normalized to pre-treatment baseline. After visual analysis, the data were transferred to “R” software, Microsoft Excel and Microcal Origin for statistical analysis and plotting of graphs.

### Western blotting

For western blot experiments we used 15 female mice randomly distributed between three treatment groups: i) group 1 received daily i.p. injections of ketamine (10 mg/kg; n = 5) for 3 consecutive days; ii) group 2 received two consecutive injections of vehicle and one injection of ketamine (10 mg/kg; n = 5); iii) group 3 received daily i.p. injections of vehicle (10 mg/kg; n = 5) for 3 consecutive days. Thus the resulting 3 groups were termed as follows: i) ketamine 72 h (n = 5); ii) ketamine 24 h (n = 5) and iii) vehicle (n = 5). On day 4 all 15 animals were euthanized with incremental CO_2_ concentration (up to 20%).

The somatosensory cortex was dissected, and immediately frozen on dry ice. The samples were sonicated on lysis buffer [20 mM Tris-HCl – pH = 8.0; 137 mM NaCl, 48 mM NaF, 1% Nonidet-40, 10% glycerol; supplemented with protease inhibitor cocktail – Roche Complete; and 2 mM Na_3_VO_4_] and centrifuged 10000xg/15 min. The supernatant was collected, the total protein levels were determined by Lowry method, and stored at −80 °C until use.

The samples (50 µg of total proteins) were resolved in SDS-PAGE gradient gels (4–12%; NuPAGE^TM^ Novex; Invitrogen; USA) and transferred to polyvinylidene difluoride membrane (PVDF). The membranes were blocked in 3% BSA-TBST [50 mM Tris, 150 mM NaCl, pH 7.6, 0.1% Tween20], and incubated with the following primary antibodies against phospho-actin (Y53; 1:1000; ECM Biosciences), PSD-95 (1:1000; Santa Cruz, #sc32290), NR2B (1:2000; Chemicon, #ab1557) and GAPDH (1:5000; Santa Cruz, #sc25778) overnight at 4 °C. Then, the membrane were briefly washed with TBST and incubated with HRP-conjugated secondary antibody (BioRad, #170–5046, #170–5047; 1:10000) for 1 hour at room temperature. The luminescence obtained after incubation with ECL was detected by a CCD camera (G:BOX, Syngene, UK). The images were analyzed using ImageJ software v1.47. The optical density for each targeted protein was normalized by GAPDH signal and expressed as percentage of the control group (*vehicle*).

### Experimental Design and Statistical Analysis

Overall, 54 mice (25 wild-type C57BL/6JRccHsd mice and 29 heterozygous B6.Cg-Tg(Thy1-YFP)HJrs/J transgenic mice obtained from the same strain of origin C57BL/6J) were used for imaging, behavioral analysis and molecular biology in this study, as summarized in the Table 1. Out of the 29 mice used for imaging, 22 mice were included in the paper based on the exclusion criteria described below.

**Table 1:** Summary of subjects used and disqualified, metrics and treatments.

mouse #	sex	date of birth	weight, g	surgery date	treatment	date of the first imaging	NS test date	WB harvest. date	Comments, attrition criteria
jk11	f	29.9.2014	15	3.11.2014	Ketamine	8.12.2014	—	—	Excluded from analysis due to lower quality of the images
jk29	f	29.9.2014	15	7.11.2014	vehicle	1.12.2014	—	—	
jk31	f	30.9.2014	16	14.11.2014	vehicle	17.12.2014	—	—	
jk38	f	27.9.2014	15	11.11.2014	vehicle	10.12.2014	—	—	Excluded from analysis due to lower quality of the images
jk39	f	27.9.2014	15	12.11.2014	Ketamine	10.12.2014	—	—	Excluded from analysis due to lower quality of the images
jk43	f	28.9.2014	15	12.11.2014	Ketamine	19.1.2015	—	—	
jk44	f	28.9.2014	16	11.11.2014	vehicle	14.1.2015	—	—	
jk46	f	30.9.2014	17	18.11.2014	Ketamine	7.1.2015	—	—	
jk61	f	11.10.2014	18	5.12.2014	vehicle	12.1.2015	—	—	
jk65	f	29.9.2014	17	5.12.2014	Ketamine	12.1.2015	—	—	
jk86	f	25.11.2014	15	29.12.2014	Ketamine	21.1.2015	—	—	
jk95	f	23.11.2014	14	23.12.2014	vehicle	21.1.2015	—	—	
jk124	f	25.11.2014	14	31.12.2014	vehicle	26.1.2015	—	—	Excluded from analysis due to lower quality of the images
jk128	f	25.11.2014	13	2.1.2015	Ketamine	28.1.2015	—	-	
jk549	f	12.5.2016	19	4.7.2016	vehicle	9.8.2016	—	—	Excluded from analysis due to lower quality of the images
jk558	f	13.5.2016	15	1.7.2016	Ketamine	9.8.2016	—	—	
jk591	f	17.5.2016	18	6.7.2016	Ketamine	16.8.2016	—	—	
jk593	f	17.5.2016	16	7.7.2016	vehicle	16.8.2016	—	—	
jk610	f	19.5.2016	20	3.8.2016	vehicle	30.8.2016	—	—	Excluded from analysis due to lower quality of the images
jk640	f	20.6.2016	19	4.8.2016	Ketamine	13.9.2016	—	—	
jk654	f	22.6.2016	19	12.8.2016	vehicle	13.9.2016	—	—	
jk690	f	28.6.2016	18	16.8.2016	vehicle	20.9.2016	—	—	
jk692	f	28.6.2016	18	16.8.2016	Ketamine	20.9.2016	—	—	
jk694	f	28.6.2016	17	18.8.2016	vehicle	20.9.2016	—	—	
jk703	f	29.6.2016	18	23.8.2016	Ketamine	27.9.2016	—	—	
jk706	f	29.6.2016	19	23.8.2016	Ketamine	27.9.2016	—	—	Excluded from analysis due to lower quality of the images
jk723	f	2.7.2016	20	29.8.2016	vehicle	4.10.2016	—	—	
jk726	f	3.7.2016	16	1.9.2016	Ketamine	4.10.2016	—	—	
jk734	f	6.7.2016	20	2.9.2016	vehicle	4.10.2016	—	—	
NS-JK378	f	7–13.12.2015	20	—	Ketamine	—	15.02–19.02.2016	—	
NS-JK379	f	7–13.12.2015	18	—	Ketamine	—	15.02–19.02.2016	—	
NS-JK380	f	7–13.12.2015	18	—	Ketamine	—	15.02–19.02.2016	—	
NS-JK381	f	7–13.12.2015	20	—	Ketamine	—	15.02–19.02.2016	—	
NS-JK382	f	7–13.12.2015	18	—	Ketamine	—	15.02–19.02.2016	—	
NS-JK383	f	7–13.12.2015	16	—	Ketamine	—	15.02–19.02.2016	—	
NS-JK384	f	7–13.12.2015	16	—	Ketamine	—	15.02–19.02.2016	—	
NS-JK385	f	7–13.12.2015	19	—	Ketamine	—	15.02–19.02.2016	—	
NS-JK386	f	7–13.12.2015	20	—	vehicle	—	15.02–19.02.2016	—	
NS-JK387	f	7–13.12.2015	18	—	vehicle	—	15.02–19.02.2016	—	
WB-NS-JK388	f	7–13.12.2015	19	—	vehicle	—	15.02–19.02.2016	11.3.2016	
WB-NS-JK389	f	7–13.12.2015	16	—	vehicle	—	15.02–19.02.2016	11.3.2016	
WB-NS-JK390	f	7–13.12.2015	18	—	vehicle	—	15.02–19.02.2016	11.3.2016	
WB-NS-JK391	f	7–13.12.2015	21	—	vehicle	—	15.02–19.02.2016	11.3.2016	
WB-NS-JK392	f	7–13.12.2015	16	—	vehicle	—	15.02–19.02.2016	11.3.2016	Excluded from WB
WB-JK393	f	7–13.12.2015	21	—	Ketamine 24 h	—	—	11.3.2016	
WB-JK394	f	7–13.12.2015	20	—	Ketamine 24 h	—	—	11.3.2016	
WB-JK395	f	7–13.12.2015	20	—	Ketamine 24 h	—	—	11.3.2016	
WB-JK396	f	7–13.12.2015	17	—	Ketamine 24 h	—	—	11.3.2016	
WB-JK397	f	7–13.12.2015	17	—	Ketamine 24 h	—	—	11.3.2016	
WB-JK398	f	7–13.12.2015	21	—	Ketamine 72 h	—	—	11.3.2016	
WB-JK399	f	7–13.12.2015	17	—	Ketamine 72 h	—	—	11.3.2016	
WB-JK400	f	7–13.12.2015	18	—	Ketamine 72 h	—	—	11.3.2016	
WB-JK401	f	7–13.12.2015	20	—	Ketamine 72 h	—	—	11.3.2016	
WB-JK402	f	7–13.12.2015	19	—	Ketamine 72 h	—	—	11.3.2016	

For animal allocation to the imaging experiments, randomization was performed prior to starting the experiments by one of the co-authors blinded to the group identity. The group allocation codes were concealed from the other co-authors performing the manual spine analysis, until the final step of data pooling and statistical analysis. The exclusion criteria for imaging experiments were as follows: animals were excluded from data analysis based on an insufficient quality of images judged by one of the co-authors performing manual spine analysis, who was blinded to the group identities. Blinding and randomization procedures used in two-photon imaging experiments in this study have been thoroughly evaluated during an on-site audit on March 22, 2016 by Partnership for Assessment and Accreditation of Scientific Practice (PAASP GmbH, Heidelberg, Germany) and were confirmed to be carried out as described in the manuscript.

For randomization of behavioral and molecular biology experiments, care was taken to allocate mice from the same group-housing cage to different treatment groups as evenly as possible.

The analysis of nest building score (n = 8 mice for ketamine group, n = 7 mice for vehicle group) was performed using a two-way ANOVA with repeated measures, having drug treatment and nest score along time as factors, and Sidak’s *post hoc* test when appropriated. The *p* < *0.05* values were considered significant.

For dendritic spine analysis (n = 11 mice for ketamine group, n = 11 mice for vehicle group), normality of the data was tested using Shapiro-Wilcoxon test of normality. In those cases when data were found to be normally distributed, comparison with 0 (baseline) was performed using Student’s t-test, otherwise Wilcoxon signed rank test was used. For the “formation”, “elimination” and “mobile fraction” parameters, we used the “greater than” version of the statistical test (because all of these values are >=0). For the “net change” parameter, the “two-sided” version of the test was used. The *p* < *0.05* values were considered significant. The comparisons between groups within a particular time interval were made using a non-parametric Mann-Whitney rank sum test. In all data analysis individual animal served as a statistical unit.

The western blotting data (n = 5 mice for vehicle group, n = 5 mice for ketamine 24 h group and n = 5 mice for ketamine 72 h group) were analyzed by one-way ANOVA followed by the Newman-Keuls or Fisher’s LSD *post hoc* tests when appropriate. The *p* *<* *0.05* values were considered significant.

### Data availability statement

The datasets generated during and/or analyzed during the current study are available from the corresponding author on reasonable request.

## Results

### Subchronic ketamine improves nest-building behavior without affecting total activity level

**Figure 1:**
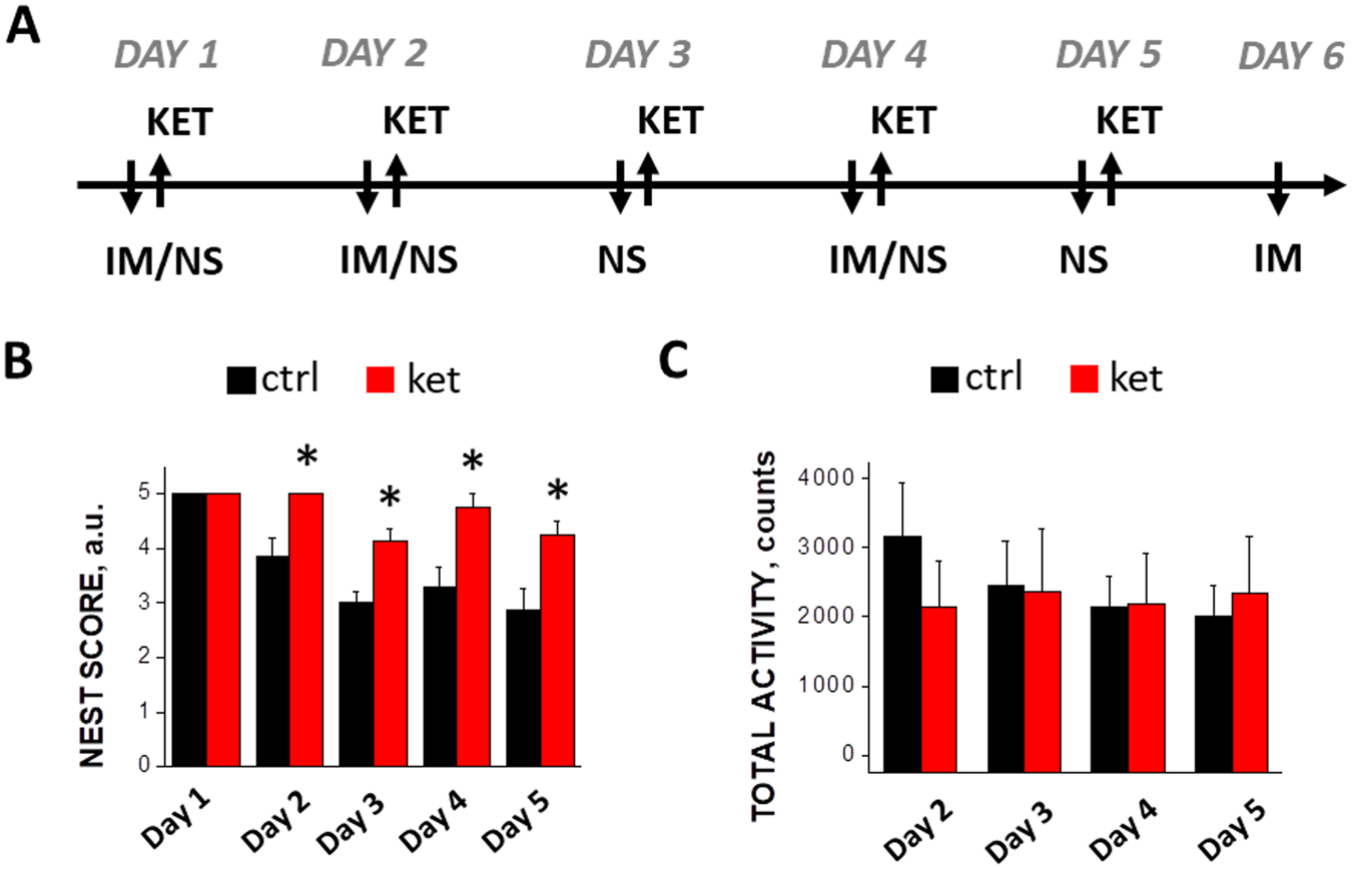
(**A**) Timeline of behavioral and two-photon imaging experiment. Abbreviations: KET – ketamine injection, NS – nest score, IM – imaging session. (**B**) Effect of ketamine treatment on nest building score, a.u. - arbitrary units; (**C**) Effect of ketamine treatment on total animal activity. See full details in Materials and methods section. *Indicates p < 0.05.

We started by analyzing the effects of the drug on the animal behavior and well-being. In the test protocol (see Fig. 1A, upper panel) ketamine was applied daily over the course of 4 days starting from Day 1, and the mouse behavior was monitored daily starting from the second day of injections. In the control mice subjected to the nest building test designed for assessment of animal well-being^22^, we observed a gradual reduction in the nest building activity. This small decline in nest score is consistent with mild deterioration in the mouse’ well-being, possibly associated with frequent handling and/or i.p. injections. Against this background, sub-anesthetic ketamine caused a sustained improvement in the nest building score, which became statistically significant starting already from Day 2, i.e. from the second day of the test, and remained at the significant level through Day 5 (see Fig. 1B). This finding suggests that repeated administration of low-dose ketamine improved well-being of single-housed mice. To exclude the possibility that this effect was due to an increased non-specific overall activity of mice, we monitored activity of the animals (see Methods for description) and found that the total activity was not affected by ketamine (Fig. 1C). Together, our behavioral data confirmed that the chosen protocol induces detectable changes in animal behavior and well-being.

### Dendritic spine turnover in somatosensory cortex under control conditions

**Figure 2:**
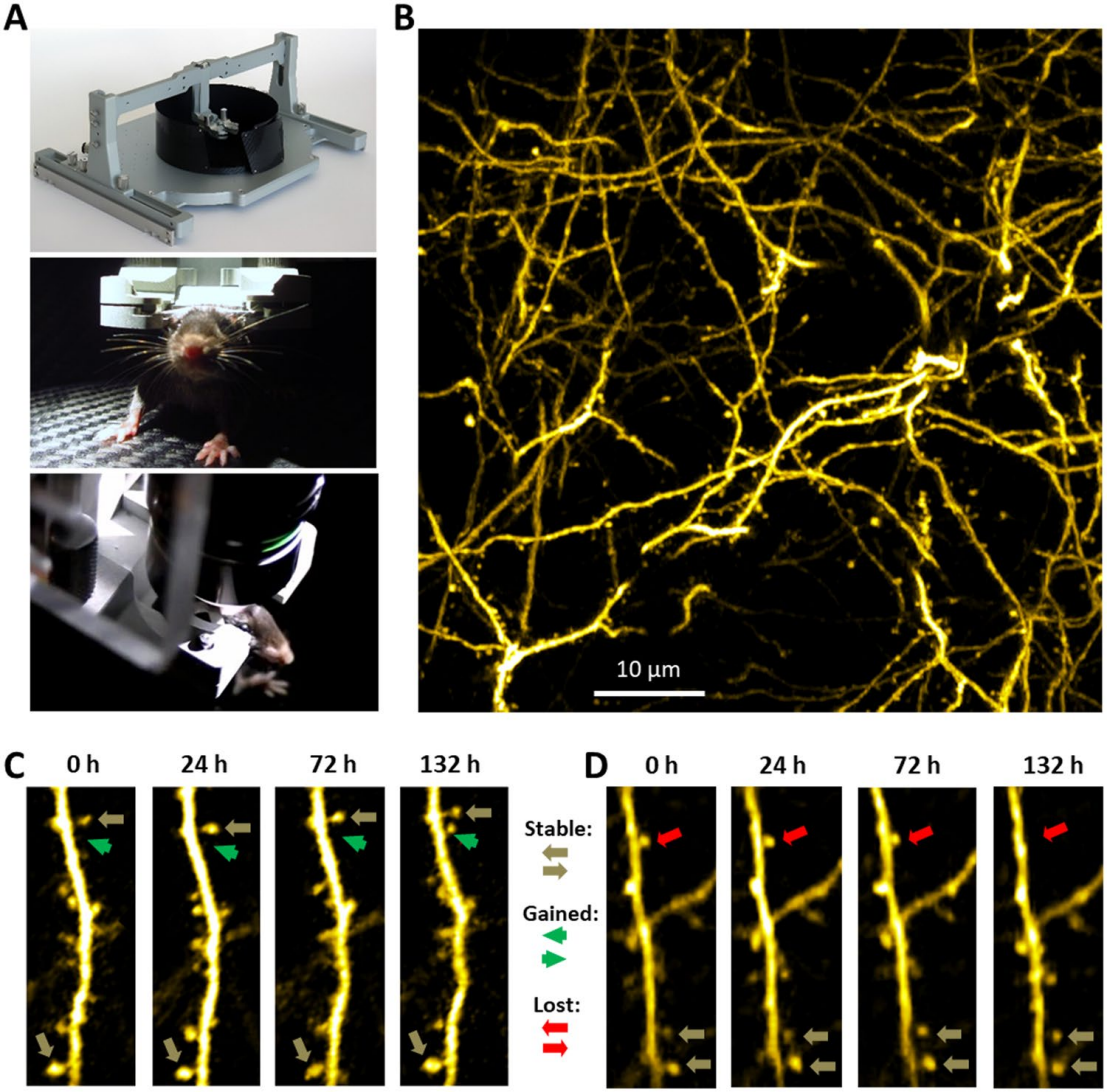
(**A**) Overview of a Mobile HomeCage platform used for head fixation of awake behaving mice (top image) and images of a mouse head-fixed in the platform under an objective during a typical two-photon imaging session (middle and bottom images). (**B**) Maximum intensity projection of 26 slices from z-stack of images showing typical architecture of layer V apical dendrites in somatosensory cortex in Thy1-YFP mice. Sequences of images showing examples of formation (**C**) of a new spine and elimination (**D**) of existing spine at 132 h time-point.

We then asked which synaptic and molecular events are associated with the observed changes in the cortex of young-adult mice. Three weeks after the implantation of cranial windows in YFP-H mice (see Methods for description of the procedure), we selected 29 animals with transparent cranial windows (Table 1) and habituated them for Mobile HomeCage device (see Fig. 2A) during 4 days of training sessions (see ref.^23^ for full description of habituation protocol). Upon image acquisition, dendritic spines were tracked blind to experimental conditions (see Fig. 2B–D for examples of spines) and statistical analysis was performed using the following four parameters: (i) formation, i.e. fraction of the spines that were newly formed (“gained”) during a given time interval (green arrow in Fig. 2C); (ii) elimination, i.e. fraction of the spines that have disappeared (were “lost”) during a given time interval (magenta arrow in Fig. 2D); (iii) net change, i.e. difference between the “formation” and “elimination” fractions, and (iv) mobile fraction, i.e. sum of the “formation” and “elimination” fractions. These four parameters are among the most commonly analyzed and presented in the publications focusing on the turnover of dendritic spines^23–25^.

**Figure 3:**
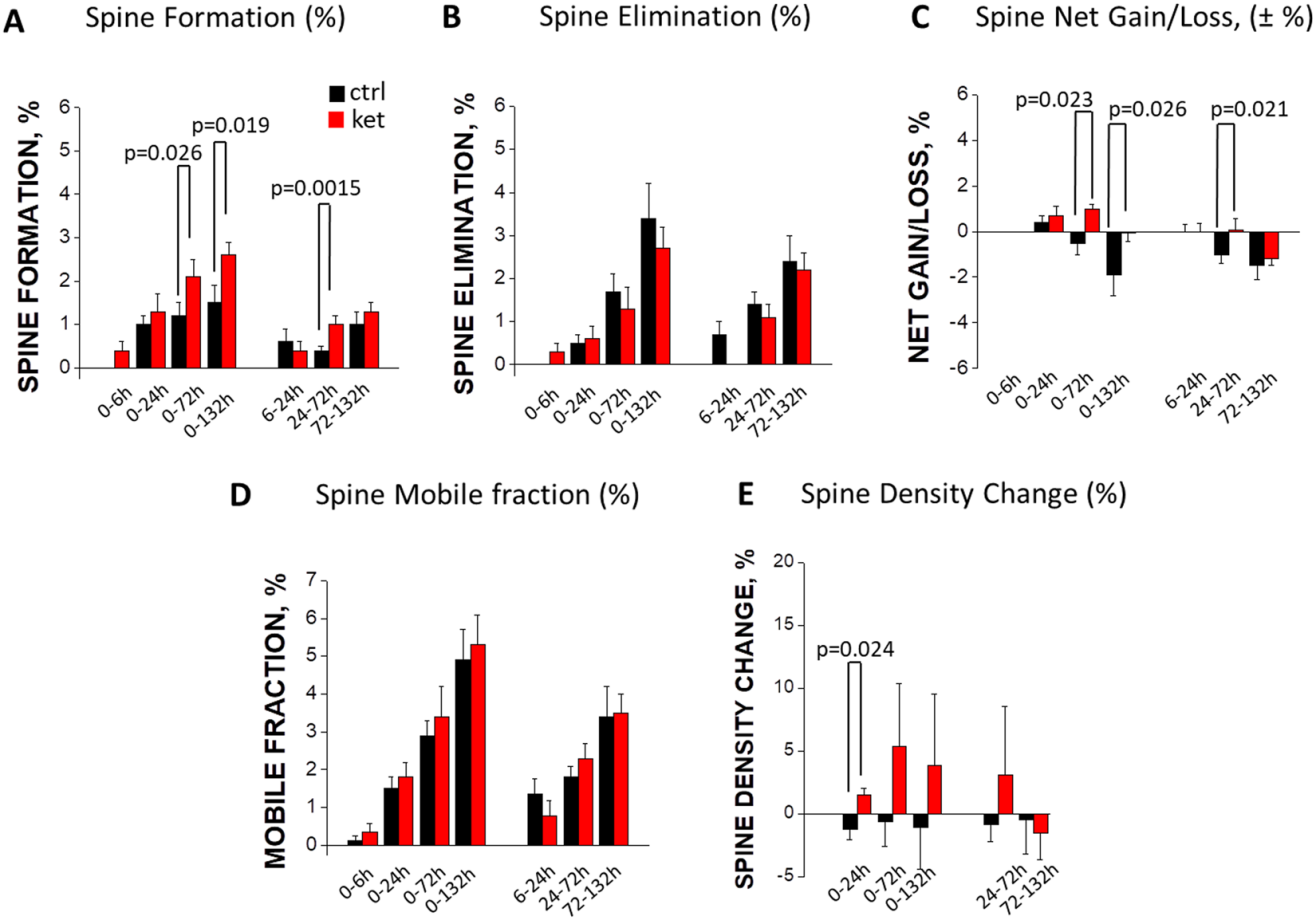
Effects of ketamine (red bars) and vehicle (black bars) treatment on spine formation (**A**), spine elimination (**B**), net gain/loss (**C**), mobile fraction (**D**) and spine density (**E**) as compared to baseline (left panel of each image) or preceding time-points (right panel of each image). Statistical P values for inter-group comparisons are shown in the Figure. See Table 2 for statistical data on comparisons with baseline (0 h timepoint).

In the control YFP-H mice, spine elimination prevailed over formation, which resulted in a statistically significant net loss of spines (1.0 ± 0.4% for the 24–72 h interval, and 1.5 ± 0.6% for the 72–132 h interval; black bars in Fig. 3C**;** see Table 2 for statistical comparison against baseline). This observation is consistent with the synapse pruning reported to occur in adolescent/young-adult mice^26,27^.

**Table 2:** Statistical data.

Data structure	Type of test	p value		
Quantification of dendritic spine turnover (n = 11 mice for ketamine group, n = 11 mice for control group), comparison with baseline(0)	Test for normality of data distribution was performed for each data set. In the right-side column of this table, only t-test values are shown for normally distributed data sets, and only Wilcoxon signed rank test values are shown for the remaining data sets.	**Vehicle treatment**		
		**Comparison group**	**t-test**	**Wilcoxon**
		Elimination 0–24 h		0.01112706
		Elimination 0–72 h	4.776974e^−4^	
		Elimination 0–132 h	8.629025e^−4^	
		Elimination 24–72 h	5.64133e^−4^	
		Elimination 72–132 h	1.653414e^−3^	
		Formation 0–24 h	9.692149e^−4^	
		Formation 0–72 h	9.277724e^−4^	
		Formation 0–132 h	8.739528e^−4^	
		Formation 24–72 h		0.010068376
		Formation 72–132 h		0.011007467
		Net 0–24 h	0.16228664	
		Net 0–72 h	0.3647341613	
		Net 0–132 h	0.06097509	
		Net 24–72 h	0.0231812	
		Net 72–132 h	0.028517536	
		Mobile 0–24 h		1.885146e^−3^
		Mobile 0–72 h		1.885146e^−3^
		Mobile 0–132 h	8.586481e^−5^	
		Mobile 24–72 h	4.043268e^−5^	
		Mobile 72–132 h	7.491859e^−4^	
		Density 0–24 h		0.371093
		Density 0–72 h	0.769134	
		Density 0–132 h	0.765564	
		Density 24–72 h	0.554025	
		Density 72–132 h	0.885468	
		**Ketamine treatment**		
		**Comparison group**	**t-test**	**Wilcoxon**
		Elimination 0–24 h		0.02952911
		Elimination 0–72 h		0.011127062
		Elimination 0–132 h	2.506081e^−4^	
		Elimination 24–72 h	3.804348e^−3^	
		Elimination 72–132 h		1.911177e^−3^
		Formation 0–24 h	2.5775378e^−3^	
		Formation 0–72 h	9.406125e^−5^	
		Formation 0–132 h	6.758756e^−6^	
		Formation 24–72 h	4.545349e^−3^	
		Formation 72–132 h	2.44514e^−5^	
		Net 0–24 h	0.13569602	
		Net 0–72 h	5.643972e^−4^	
		Net 0–132 h	0.91243574	
		Net 24–72 h	0.8663546	
		Net 72–132 h	1.866831e^−3^	
		Mobile 0–24 h	7.750874e^−4^	
		Mobile 0–72 h	5.193477e^−4^	
		Mobile 0–132 h	2.294916e^−5^	
		Mobile 24–72 h	3.520205e^−5^	
		Mobile 72–132 h	8.882077e^−6^	
		Density 0–24 h		0.100348
		Density 0–72 h	0.332934	
		Density 0–132 h	0.529371	
		Density 24–72 h	0.590639	
		Density 72–132 h	0.498503	

### Subchronic ketamine administration boosts spine formation

We used two common ways of quantifying the spine dynamics: 1) cumulative (i.e., change in spines between the pre-treatment baseline and a certain time point), and 2) inter-session (i.e., change in spines between two subsequent imaging sessions). The analysis of the effects of ketamine on spine formation (“gain”) showed that, using both cumulative and inter-session metrics, there was significantly more newly-formed spines in ketamine-treated group compared to the control animals (Fig. 3A). Thus, in the control group there were only a few newly formed spines (around 1.5% cumulatively for 0–132 h; p < 0.001 relative to baseline; black bars in Fig. 3A), and the ketamine treatment nearly doubled the spine formation (2.6 ± 0.3% cumulatively for the 0–132 h; p < 0.001 as compared to the baseline, and p = 0.019 as compared to the control group; red bars in Fig. 3A). The inter-session analysis confirmed that spine formation rates were significantly higher in ketamine-treated mice compared to control (1.0 ± 0.2% versus 0.4 ± 0.1% for the 24–72 h interval; p = 0.0015, Fig. 3A). These data demonstrate that low-dose subchronic ketamine is a potent stimulator of dendritic spine formation.

### Subchronic ketamine has no effect on spine elimination

As mentioned above, in the vehicle-treated group, the spine elimination (“loss”) was more pronounced than spine formation. We calculated the effects of the subchronic ketamine treatment on spine elimination (Fig. 3B), and found that the continuous spine elimination was very similar between the control and ketamine-treated groups throughout the whole duration of the imaging experiments (132 h). Thus, cumulative spine elimination in both vehicle-treated and ketamine-treated mice during the 0–72 hour interval was significantly different from the baseline (see Table 2), but the there was no difference between these groups in terms of spine elimination (p = 0.2143; Fig. 3B). These data indicate that subchronic low-dose ketamine has no effect on spine elimination in mouse somatosensory cortex.

### Ketamine treatment reverses net loss of spines and induces net gain

A common way of quantifying spine turnover is calculating the net effect of spine formation and elimination, by subtracting the percentage of “lost” spines from the percentage of “gained” spines. The resulting values, when positive, represent a “net gain” of spines, which is characteristic of the rapid synaptogenesis observed during the early postnatal period; when negative, the net values represent a “net loss” typical for the adolescent/young-adult animals undergoing synapse pruning; the near-zero values correspond to the “net equilibrium” observed in adult animals.

The cumulative net changes in spines are shown in Fig. 3C (left panel), and the inter-session net changes are shown in Fig. 3C (right panel). The vehicle-treated group demonstrates a strong trend towards the net loss of spines over the course of our study, reaching a near-significant level by 132 h (p = 0.061 for 0–132 h, see Table 2). The net change in the control group was found to be significantly different from the baseline at the 24–72 h time and at 72–132 h interval (p = 0.023 for 24–72 h and p = 0.029 for 72–132 h, Table 2). Ketamine treatment reversed the net loss of spines observed in the control group; instead, a significant net gain of spines was observed in the ketamine group during the 0–72 h time interval (Table 2). The differences between the two groups were statistically significant (p < 0.05 for three time intervals: 0–72 h, 0–132 h and 24–72 h; Fig. 3C).

### Ketamine does not affect the mobile (unstable) fraction of spines

Calculating the “mobile fraction” is another way of assessing spine turnover, which quantifies the percentage of spines that are unstable (i.e., are either lost or gained during a given time interval). This metrics allows the assessment of a “destabilizing”, or “mobilizing”, effect of a treatment on dendritic spines. Our calculations of the mobile fraction show that the percentage of unstable spines was statistically indistinguishable between the vehicle- and ketamine-treated groups (Fig. 3D). Cumulatively, both groups showed the mobile fraction values around 3–5%, which were significantly different from the pre-treatment baseline (p < 0.01 beginning from the 0–24 h time interval; Table 2) but not different between the groups (p > 0.05 for all time-points; Fig. 3D).

### Ketamine transiently increases spine density

In addition to spine turnover, we also calculated the spine density at each imaging time point (Fig. 3E). Interestingly, two groups showed opposite trends: ketamine-treated mice tended to increase their spine density, while in vehicle-treated mice the density tended to decrease. However, spine density in both vehicle-treated and ketamine-treated mice was not significantly different from the baseline (see Table 2), but the there was a statistically significant difference in terms of spine density between these groups during the 0–24 h interval (p = 0.024; Fig. 3E). This is consistent with our finding that subchronic ketamine reverses net loss of spines observed in vehicle-treated mice (Fig. 3C).

Taken together, the spine imaging data demonstrate that, under the control conditions, spine elimination prevails over spine formation, resulting in net loss of spines, and that subchronic ketamine treatment increases spine formation, resulting in net equilibrium and, at some time points, net gain of spines. Consistently, ketamine increases spine density but does not affect the size of mobile fraction of spines.

### Ketamine treatment elevates protein levels for phospho-actin and PSD-95 but not NR2B

**Figure 4:**
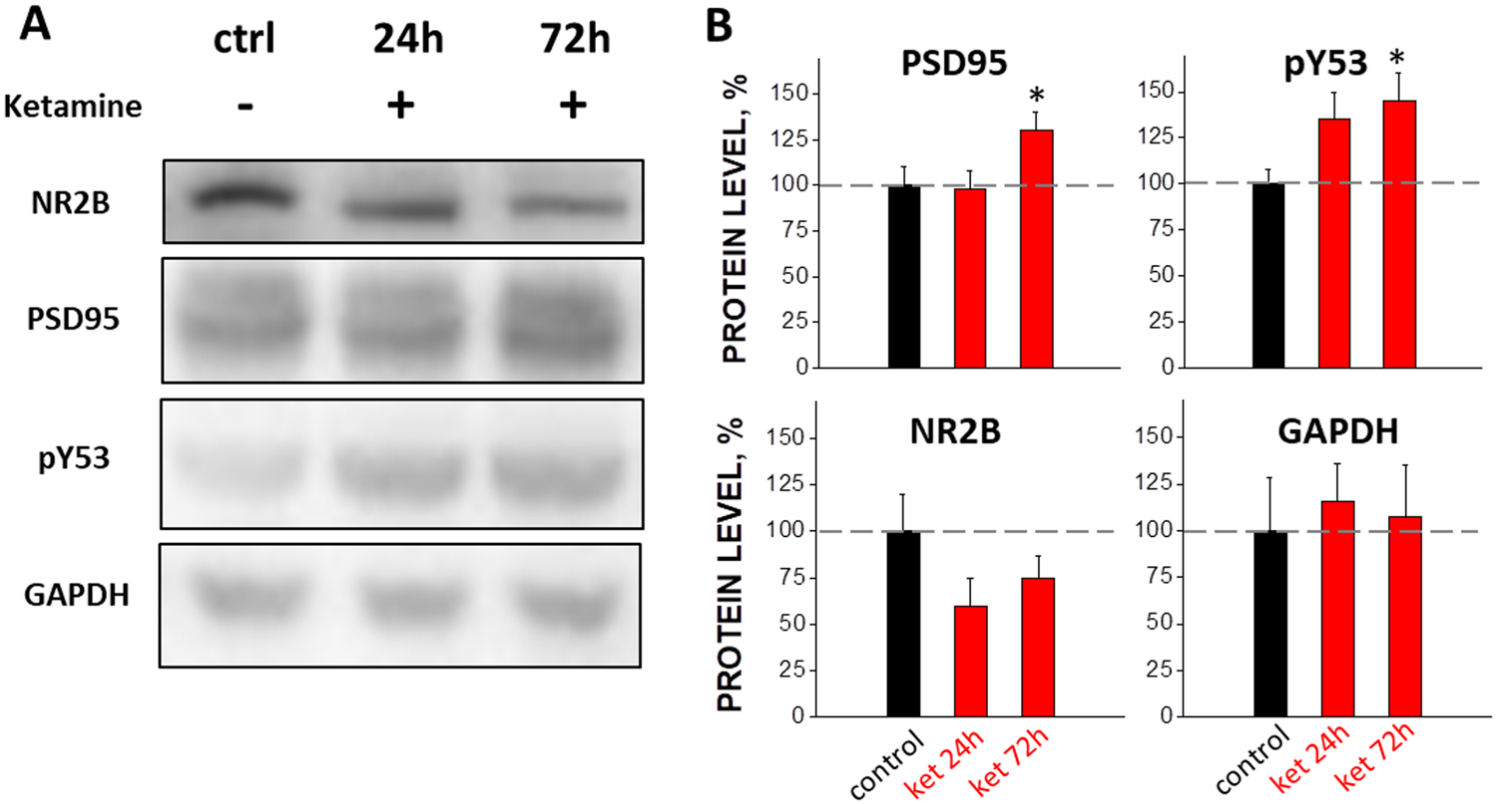
Western blot images (**A**) and summary of effects of ketamine on the expression level of PSD-95, pY53 and NR2B (**B**) in somatosensory cortex at 24 and 72 hours after the start of subchronic treatment. *Indicates p < 0.05.

To elucidate possible molecular mechanisms underlying the stimulating effects of ketamine on spine formation, we analyzed *ex vivo* brain tissues harvested from somatosensory cortices and measured the level of key proteins associated with synaptic plasticity. Pharmacologically, the primary mechanism of ketamine action is its non-competitive antagonism of NMDA-type glutamate receptors^28^. Antagonists of the NR2B subunit of the NMDA receptors have been shown to possess antidepressant-like activity comparable to ketamine^29^ and knockdown of NR2B mimics antidepressant effects of ketamine^30,31^. We observed a trend towards reduced expression of NR2B subunit in ketamine-treated mice; however, this effect did not reach significance (p > 0.05; Fig. 4).

It was shown earlier that brief over-expression of an actin phosphorylation-mimicking mutant construct increased the dynamics of dendritic spines^32^. At the same time, over-expression of the phospho-mimicking actin construct increased the sizes of synapses, on both pre- and postsynaptic sites, suggesting that actin phosphorylation facilitated synapse formation. We measured the level of actin phosphorylated on residue Y53 and found that it increased 33 ± 11% by 24 hours and 47 ± 13% by 72 hours after the first ketamine injection (Fig. 4).

PSD-95 is known to be associated with mature excitatory synapses^33^. Our Western blot analysis showed that the expression level of PSD-95 increased by 72 hours (i.e. at the time point where we observed significant effects of ketamine on spine turnover) after the first ketamine injection (28 ± 8% increase at 72 h, p < 0.05; Fig. 4).

Our findings of increased PSD-95 and phosphorylated actin, taken together with the boost in dendritic spine formation, strongly suggest that subchronic ketamine administration induces sustained changes in synaptic plasticity.

## Discussion

To the best of our knowledge, this is the first study quantifying the effects of subchronically administered ketamine on spine turnover in awake, behaving mice. The main finding of this study is that repetitive administration of ketamine induced a sustained shift in dendritic spine turnover towards spine formation and promoted synaptogenesis by increasing phosphorylated actin (pY53) and elevating PSD-95 expression. Taken together, our biochemical and anatomical data complement each other in demonstrating that (and suggesting how) subchronic ketamine induces synaptic plasticity in somatosensory cortex.

Recent clinical studies indicated that repeated ketamine infusions achieved superior outcomes as compared to a single infusion^16,34,35^. The preclinical data presented here support the utility of repeated ketamine infusions for greater efficacy in clinical settings.

The present study was designed to test both general effects of ketamine on the animal’s wellbeing and behavior as well as its effects on cellular and molecular mechanisms. Nest building activity is an indicator of health and welfare in laboratory mice^36^. The improvement in the nest building score we observed with subchronic ketamine administration suggests the general positive influence of this treatment on laboratory mice. However, the observed effect of ketamine cannot be extrapolated to any specific pathological condition, because the nest building test is not generally used as an assay for depression or stress.

The present data also show that the structural remodeling observed previously with ketamine treatments^10^ is not limited to frontal/prefrontal cortex. A comparison between our study and the recently published *in vivo* data from the medial frontal cortex^11^ highlights both similarities and differences. On the one hand, in both cases ketamine elevated the spine formation rate; on the other hand, in our study the stimulating effect of ketamine on spine formation in the somatosensory cortex reached significance only 72 hours after the first injection (Fig. 3), while in the frontal cortex ketamine’s effect was significant already at 24 hours^11^. This comparison suggests a faster rate of development of the effect of systemic ketamine in the frontal cortex *versus* somatosensory. However, one should also take into account the methodological differences between the two studies: namely, single injection of ketamine *versus* subchronic administration, and imaging in anesthetized *versus* awake animals.

An important advantage of the present study is that it was performed under the conditions of minimal invasiveness (chronic cranial windows over somatosensory cortex) and maximal physiological relevance (longitudinal imaging in awake behaving mice). To overcome limitations of general anesthesia and to avoid the negative side-effects of the stress associated with full-body animal restraint, we used a recently developed air-lifted platform for imaging the brain of head-fixed but otherwise freely moving and behaving animals^37,38^. We started spine imaging no sooner than 3–4 weeks after the cranial window surgery, and selected only those mice that had transparent windows and showed no signs of residual post-surgical inflammation (see the Standard Operation Procedures described in the Methods section). The somatosensory cortex area was selected for several reasons: a) it is not known whether synaptogenic effects of ketamine occur beyond frontal/prefrontal cortex; b) somatosensory cortex can be imaged *in vivo* without a major surgery that may cause substantial damage to brain tissue (as occurs with glass prism implantation); c) several studies have shown that dissociative effect of ketamine is accompanied by a functional disruption of cortico-cortical pathways^39,40^, which may lead to restructuring of synaptic pathways in somatosensory cortex; d) being a primary site for decoding proprioceptor and nociceptor information^41^, somatosensory cortex may serve as a relevant area for ketamine-induced plastic changes in the brain.

Another feature of our study consists in the special efforts that were made to minimize possible biases during both imaging and data analysis. First, mice of only one gender were used in this study. Second, mice were carefully selected for imaging based on the transparency of their cranial windows. Third, the selected mice were randomized before the start of the imaging procedure. Fourth, the researcher performing analysis of individual dendritic spines was blinded to the identity of the treatment groups; the whole image sequence data were analyzed by one person to minimize possible inter-person variability in the spine counting method. Finally, individual animals, rather than spines or imaging areas, served as experimental units for statistical analysis.

However, this study has several limitations. First, imaging was limited to one week, and possible long-term changes in the spine turnover were not studied after the cessation of ketamine treatment. Second, while this study expands our knowledge on synaptogenic effects of ketamine beyond prefrontal cortex, we had to limit the scope of our experiments to only one cortical region (somatosensory). Third, the limited size of this study did not allow us to compare the effects of a single dose of ketamine with those of subchronic administration, which should be addressed in future studies.

This study was performed in young adult mice (2–3 months of age) that are known to undergo synaptic pruning, i.e. at the age when in layer 5 pyramidal neurons of somatosensory cortex elimination of synapses prevails over their formation^26,27^. The re-balancing effect of ketamine on synaptic inputs may underlie some of the complex behavioral effects of ketamine and warrants further investigation.
